# Time-dependent Bivariational Principle: Theoretical Foundation for Real-Time Propagation Methods of Coupled-Cluster Type

**DOI:** 10.1021/acs.jpca.4c07417

**Published:** 2025-04-06

**Authors:** Simen Kvaal, Håkon Richard Fredheim, Mads Greisen Højlund, Thomas Bondo Pedersen

**Affiliations:** † Hylleraas Centre for Quantum Molecular Sciences, Department of Chemistry, University of Oslo, P.O. Box 1033 Blindern, Oslo N-0315, Norway; ‡ Department of Chemistry, Aarhus University, Langelandsgade 140, Aarhus C 8000, Denmark

## Abstract

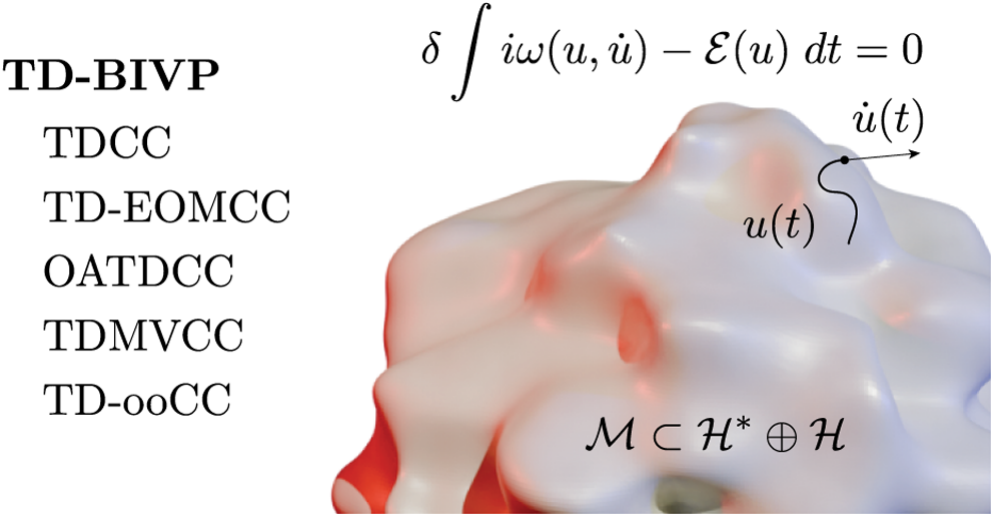

Real-time propagation methods for chemistry and physics are invariably formulated using variational techniques. The time-dependent bivariational principle (TD-BIVP) is known to be the proper framework for coupled-cluster type methods, and is here studied from a differential geometric point of view. It is demonstrated how two distinct classical Hamilton’s equations of motion arise from considering the real and imaginary parts of the action integral. This in turn leads to two distinct bivariational principles for real bivariational approximation submanifolds. Conservation laws and Poisson brackets are introduced, completing the analogy with classical mechanics. Furthermore, the time-dependent univariational principles (the time-dependent variational principle, the McLachlan principle, and the Dirac–Frenkel principle) are reconstructed using the TD-BIVP and a bivariational submanifold on product form. An overview of established real-time propagation methods is given in the context of our formulation of the TD-BIVP, namely time-dependent traditional coupled-cluster theory, orbital-adaptive coupled-cluster theory, time-dependent orthogonal optimized coupled-cluster theory, Brueckner coupled-cluster theory, and equation-of-motion coupled cluster theory.

## 1 Introduction

With the increase in demand for high-accuracy first-principles simulations of the quantum dynamics of molecular systems at the attosecond time scale comes the need for theoretical frameworks that allow derivation of affordable, accurate, and systematically improvable computational tools. The cornerstone of approximate quantum dynamics has for more than half a century been the Dirac–Frenkel variational principle (DFVP), the time-dependent variational principle (TDVP) and the McLachlan variational principle (MVP), often collectively referred to as the Dirac–Frenkel–McLachlan variational principle, even though they are in general not equivalent.^1−8^ They are all time-dependent generalizations of the Rayleigh–Ritz variational principle. However, the most popular wave function-based method in quantum chemistry is the coupled-cluster (CC) method, which is notable for being not variational—it is bivariational.

In this article, we present a comprehensive study of the time-dependent bivariational principle (TD-BIVP)^9,10^ which generalizes the TDVP to Hamiltonians that are formally not assumed to be Hermitian, and forms the proper setting for the various forms of time-dependent CC theory that have been developed over the last decades; from time-dependent traditional CC theory,^11,12^ via equation-of-motion CC theory,^13,14^ to orbital-adaptive and orbital-optimized CC theory.^15,16^ Applications of time-dependent CC theory with a bivariational formulation covers applications as diverse as electronic-structure theory,^11,17^ the vibrational Schrödinger equation,^16,18−20^ and nuclear structure theory.^21^ For a recent review, see ref (22). Considering the importance of variational principles for the development of computational tools on one hand, and the growing importance of time-dependent CC theory on the other, establishing the theoretical framework of the TD-BIVP can therefore catalyze rapid progress in the development of sophisticated and relatively low-cost real-time propagation methods in several fields, and in particular in attochemistry.

The TD-BIVP was first mentioned in passing by Chernoff and Marsden,^9^ without coining the term, who devised a Lagrangian density (in the sense of field theory), and a corresponding action , in which the system wave function and its complex conjugate were formally independent variables ψ and ψ̃ forming canonically conjugate variables in an abstract phase space . The Euler–Lagrange equations were the time-dependent Schrödinger equation and its dual, written as a pair of Hamilton’s equations of motion on complex form. The principle was independently discovered by Arponen in his seminal treatise on coupled-cluster (CC) theory,^10^ where the CC amplitudes turn out to be canonical variables, preserving the form of Hamilton’s equations of motion. In later publications by the trio of Arponen, Bishop and Pajanne on the extended CC method, the principle was occasionally invoked, emphasizing the canonical structure of these methods, including small oscillations around the ground state solution.^23−27^ A more formal symplectic geometry formulation of coupled-cluster theory was first considered by Arponen,^28^ who identified a real Hamiltonian system; see Section 2.1 in this article.

While the canonical transformation of time-dependent CC theory is an exact reformulation of quantum dynamics, approximations are invariably introduced in the form of the conventional hierarchy of CC with singles, doubles, triples, etc. Such approximations amount to choosing a submanifold  of phase space and restricting variations of the variables in the action  to be in the tangent space of , in a similar fashion as is done for the Dirac–Frenkel and McLachlan principles.^5^ Traditionally, the manifold  has been assumed to be complex in the bivariational case,^11,15,18,29^ i.e., the local coordinates are complex numbers and the points on  are complex differentiable with respect to the coordinates. The argument has been that since  is complex-valued, the manifold must be complex in order to give well-defined equations of motion. In this article, we show that this can be refined, and a careful consideration of real manifolds yields two distinct time-dependent bivariational principles,  and  while the original principle becomes ill-defined as complex differentiability is no longer true. This is analogous to the well-known relationship between the various time-dependent univariational principles:^5,30^ DFVP is formulated for complex manifolds only, and the TDVP and MVP principles are obtained as, respectively, imaginary and real parts of the DFVP. When the manifold is complex, TDVP and MVP become equivalent to the DFVP, while for real manifolds, the principles are distinct, and the DFVP is ill-defined.

Indeed, the analogy can be made stronger: We prove that univariational theory can be canonically extended to a bivariational formulation, where  is equivalent to the DFVP,  is equivalent to the TDVP, and  is equivalent to the MVP.

For examples beyond univariational theories, the principle  is exemplified by the family of orbital-optimized time-dependent CC methods (TD-ooCC) discussed by Sato and co-workers,^31,32^ as well as the split time-dependent modal vibrational CC method (sTDMVCC) introduced by Højlund and Christiansen,^33^ while the second variational principle  has not been put to use, to our knowledge. Indeed, it is an interesting open question for which submanifold approximations the different principles are meaningful.

The remainder of this article is structured as follows. In Section 2 we introduce the TD-BIVP, with emphasis on symplectic geometry. In Section 3 we consider the restriction of the bivariational dynamics to (symplectic) submanifolds, and develop the equations of motions in local coordinates. We consider both real and complex submanifolds. We also discuss the relation of the TD-BIVP to the univariational principles. In Section 6, we formulate bivariational dynamics methods in the literature using the present abstract framework. Finally, in Section 7 we present our conclusion and future perspectives. An Appendix provides additional details.

## 2 Time-dependent Bivariational Principle

The time-dependent bivariational principle is a stationary-action principle for the time-dependent Schrödinger equation and its dual. Consider the action-like functional
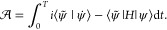
1

Here, , a wave function in a complex (separable) Hilbert space, and , an element of the complex-conjugate Hilbert space, or dual space.^34^ The notation ⟨·|·⟩ is thus the dual pairing on  (Note that ψ̃ is *not* meant to be the complex conjugate of ψ, but an independent variable that lives in dual space.) In physics terms,  is the space of “kets”, while  is the space of “bras”. Note that we do not interpret ⟨·|·⟩ as the inner product, which is sesquilinear and hence not complex differentiable. The system Hamiltonian *H* is assumed (for simplicity) to be a bounded operator over . However, *H* does not have to be self-adjoint in general since ψ̃ and ψ are independent.

The action is stationary  under arbitrary smooth variations (vanishing at the end points) of ψ and ψ̃ if and only if
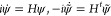
2i.e., the action principle is equivalent to the time-dependent Schrödinger equation and its dual. Here,  =  in bra–ket notation is called the Banach adjoint, or operator transpose.^35^ With the Hamiltonian function  = , eq 2 takes the form of a complex set of Hamilton’s equations of motion
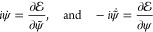
3which comes as no surprise when  is recognized as the *Modified Hamilton’s Principle* from classical mechanics.^36^ Thus,  serves as phase space, with  forming a pair of (infinite-dimensional) momenta and coordinate vectors, respectively.

The action  implies two conserved quantities: First, the overlap is conserved, , and second, the energy is conserved, .

We take note of a small curiosity, also present for the Modified Hamilton’s Principle for classical dynamics:  has a critical point only if  are *actually* solutions of eq 3 with initial conditions . On the other hand, the variations do not “see” the boundary conditions, since they are supported in the interior of the time interval [0, *T*]. Thus, the action functional can be viewed as a semilocal integral formulation of the time-dependent Schrödinger equation, and we will omit the specification of the time boundaries when writing the action integrals.

In exact quantum mechanics and in the TDVP, we have *H* = *H*^†^, and the only allowed initial conditions satisfy  = , and consequently  =  for all *t*. (In general,  is the injection of  into complex-conjugate space.)^34^ However, the power of the bivariational principle is that it allows far more flexible approximation schemes than the TDVP, since we may allow ψ̃ and ψ to have independent approximations, such as is the case in coupled-cluster theory.

### 2.1 Complex Hamiltonian Systems as Real Hamiltonian Systems

Complex Hamiltonian equations of motion may seem strange and very different from the standard real Hamiltonian systems of classical mechanics. In this section we demonstrate, however, that the complex Hamiltonian system is in fact just real Hamiltonian systems in disguise.

The real and imaginary parts of the action  must be simultaneously stationary if . Following the idea of Arponen^28^ we show that the complex Hamiltonian system is in fact equivalent to a standard real-valued Hamiltonian system: Let  and  be biorthogonal bases, i.e.  = , and define real-valued vectors *q*_*i*_ and *p*_*i*_, *i* ∈ {1, 2}, such that
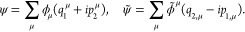


We obtain, up to a total time derivative
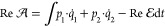
4aand
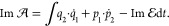
4b

We recognize the real part of  as the Lagrangian from the Modified Hamilton’s Principle,^36^ and consequently
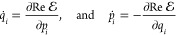
5a

For the imaginary part, set (*Q*_1_, *Q*_2_) = (*q*_1_, *p*_2_) and (*P*_1_, *P*_2_) = (*q*_2_, *p*_1_) to obtain another Hamiltonian system
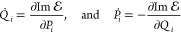
5b

Since  is complex differentiable, eqs 5a and 5b can be seen to be related by the Cauchy–Riemann equations, and hence equivalent. We make the observation that the complex Hamiltonian system (3) is equivalent to two *distinct* standard real Hamiltonian systems. Hamiltonian systems with multiple distinct symplectic structures are called bi-Hamiltonian systems.^37^

### 2.2 Symplectic Formulation of the Bivariational Principle

We now develop the theory of the bivariational principle using a more abstract approach. This will in the end produce very concise and informative expressions. We equip our phase space  with a symplectic form ω:  → , an antisymmetric and nondegenerate bilinear map, which we define using the expression
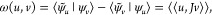
where , , and  ≡ . The notation ⟨⟨*u*, *Jv*⟩⟩ is the dual pairing of  =  ⊕  and  =  ⊕ , and does *not* denote the Hermitian inner product on . In fact, we will never use an inner product on . (The reader may note that in this particular dual pairing—natural from the context—the dual element is to the *right*, as opposed to the left as in ⟨·|·⟩.) Consequently,  is now a (linear) symplectic space.

For an operator *A*: , let us introduce the “symmetrization” *Â*:  as
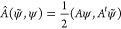
6and the corresponding bilinear functional  =  = . We define the notation  ≡  = . The action functional reads, up to a total time derivative
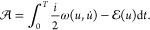
7

Let  be an arbitrary variation. Since ω is antisymmetric, and  is symmetric, integration by parts readily yields
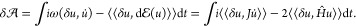
8Here,  is the Fréchet derivative of , and  =  is correspondingly the directional derivative of  in the direction δ*u*. Since δ*u* was arbitrary, Hamilton’s equations of motion (3) become
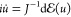
9which, due to the special form of  reduces to
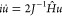
10which is equivalent to eq 2.

## 3 Evolution on Manifolds

In this section we describe the bivariational evolution on submanifolds of phase space. We first deal with complex manifolds, where complex differentiation can be used, and then with the more general real manifolds, which will allow us to identify two distinct time-dependent bivariational principles which reduce to the complex case under certain conditions.

### 3.1 Complex Manifolds

**Figure 1:**
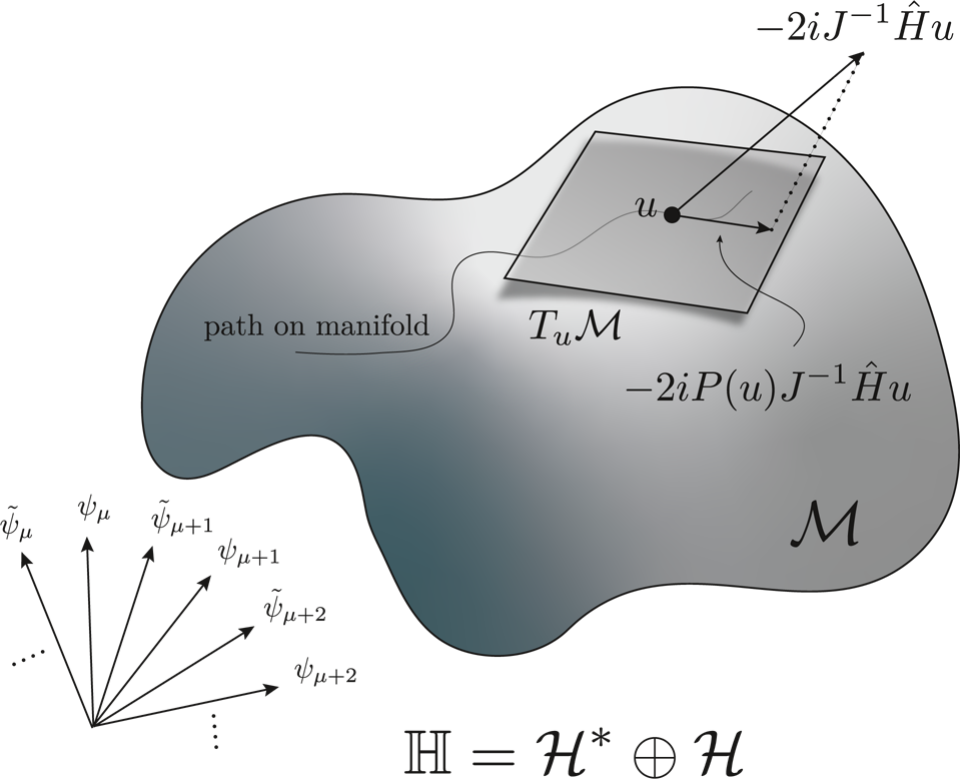
Illustration of infinite dimensional phase space, a submanifold , and the symplectic projection that dictates time evolution on the manifold.

Approximate time evolution is obtained from the bivariational principle by introducing a smooth submanifold  and restricting the principle of stationary action to ; see Figure 1 for an illustration. We assume for simplicity that  is a complex manifold of finite dimension *n*.

Let . Let  be an arbitrary variation. We obtain for the variation of the action
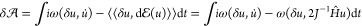
11and the corresponding Euler–Lagrange equation
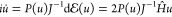
12where  is the *symplectic projection* at *u*: For any , the projection  ∈  is defined by the condition
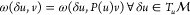
13

The symplectic projection *P*(*u*) is well-defined whenever the restriction ω:  ×  →  of the symplectic form is nondegenerate, which in turn is the definition of  being a symplectic submanifold of .

### 3.2 Real Manifolds

Suppose now  is a *real* manifold of finite dimension *m*. Then, if we consider the functionals
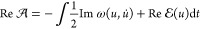
14a
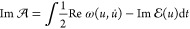
14bit no longer holds in general that  = 0 and only if  = 0. This suggests that we can, in principle, generate *different* approximate time evolutions on  from each real-valued action.

The forms Re ω and Im ω are antisymmetric nondegenerate bilinear forms on a real phase space we will denote : There is a standard way to view a complex linear space  as a real linear space , called the realification of , by restricting the field of multiplicative scalars to . The set of vectors is the same. Multiplication *u*→*iu* still yields an element of , but now as a linear operator **i** that satisfies **i**^2^ = −Id (Such an operator is called a complex structure on the vector space.) In particular, *u* and i*u* are linearly independent in , and the dimension of the space is therefore doubled. Moreover, suppose a nondegenerate bilinear form *a*:  ×  →  is given. Both Re *a* and Im *a* are now nondegenerate bilinear forms on . If *a* is symmetric/antisymmetric, then Re *a* and Im *a* are also symmetric/antisymmetric. We conclude that  and  are distinct symplectic linear manifolds.

Select a *real* submanifold , and find, in a similar manner as previously, that  = 0 for all infinitesimal variations  ∈  if and only if
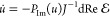
15awhere *P*_Im_(*u*) is the symplectic projection operator onto the *real* tangent space  obtained from the symplectic form Im ω. Similarly,  for all infinitesimal variations if and only if
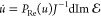
15bwhere *P*_Re_(*u*) is the symplectic projection obtained from the symplectic form Re ω. The existence of the symplectic projection depends on the invertibility of its matrix in the tangent basis; see Section 3.4. Equivalently, we must require that  is a symplectic submanifold, where the symplectic form is nondegenerate by definition.

The equations of motion (15a and 15b) are explicitly real equations of motion, and, in the case , equivalent to the canonical equations of motion (5a and 5b). For general submanifolds, however, the Cauchy–Riemann equations do not apply, and the two Euler–Lagrange equations are not equivalent, i.e., they generate distinct time evolutions on  whenever they apply. On the other hand, it can happen that  is simply a re-expression of a complex manifold using real and imaginary parts of the complex coordinates. In that case, the Cauchy–Riemann equations again apply, and  and  are equivalent functionals, i.e., eq 15a and 15b generate the same time evolution on .

### 3.3 Manifold Normalization

A word on normalization of bivariational approximation manifolds  is in place. Implicit in eq 1 is the assumption that , and this normalization is preserved by the time evolution. Indeed, the action  can be derived from a more fundamental action principle, see the Appendix, written explicitly on a phase and normalization invariant form using the bivariational density matrix
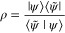


If the manifold  either satisfies  = 1 everywhere, or if  contains phase and normalization scalings  ∈  for any fixed  ∈ , then the two principles of stationary action give the same solutions. In practice, such normalized or scale invariant manifolds are always easy to construct, given a manifold that does not initially satisfy the constraint.

### 3.4 Euler–Lagrange Equations in Local Coordinates

We express the complex and real Euler–Lagrange equations in terms of local coordinates, beginning with the complex case. Let  ⊂  be a complex submanifold of dimension *n* < +∞, for simplicity. Let *u* ∈  ⊂  be given in local coordinates by *u* = Φ(*z*), with  ∈ , and let  =  =  =  define the coordinate basis vectors. By assumption, this is a linearly independent set. Any tangent vector δ*u* ∈  is now expanded as δ*u* = ∑_μ_*t*_μ_δ*z*^μ^. The equations of motion are readily obtained by considering eq 11 which leads to the Euler–Lagrange equation
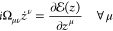
16where
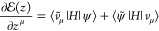
and where
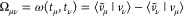
17is a complex antisymmetric matrix. We use the Einstein summation convention. We note that the antisymmetry implies that (the complex dimension)  = *n* must be even. The matrix Ω is invertible over the manifold if and only if  is a symplectic submanifold.

Inverting Ω in eq 16, multiplying with *t*_ν_ and summing, we obtain the following representation of the symplectic projection operator
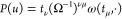
18

The calculation of the Euler–Lagrange equations for the real manifold case is very similar. We let  be a real submanifold of dimension *n* < +∞, and denote as before  =  ∈ , the coordinate basis for tangent space at *u* =  ∈ , where now *x* = (*x*^μ^) are real-valued local coordinates. We obtain, for  = 0 and  = 0, respectively
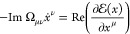
19aand
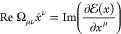
19b

In both cases, the coefficient matrix is a real antisymmetric matrix. It follows that  = *n* must be even both cases. Whenever the matrix inverses exist over the manifold,  and  are real symplectic manifolds, which must be of even (real) dimension. By a similar argument as for eq 18, we derive the following representations for the real symplectic projection operators
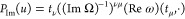
and
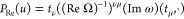


### 3.5 Interpretation

Taking the real part of  to define an action functional is not a novel idea, and dates back at least to Pedersen and Koch.^38^ The family of TD-ooCC methods^32^ as well as the split TDMVCC^33^ method for vibrational dynamics (see also Section 6) are defined in terms of taking the real part of a noncomplex differentiable action. Indeed, for bivariational methods such as the coupled-cluster method, it is a well-known problem, or feature, that observables may attain nonzero imaginary values, even if *H* = *H*^†^. It is customary to simply insist on taking the real part of the computed observable, and discard the (hopefully) small imaginary parts. This would be equivalent to using the action  and using the Hellmann–Feynman interpretation of expectation values.^38^ Indeed, for , the real part  of the energy expectation value is now the generator for the dynamics, and for a generic observable *O*, the expectation value functional reads  = , where *Î* is the symmetrization of the identity operator.

On the other hand, we have also found that taking the *imaginary* part  yields a distinct approximation when  is real. Then,  is the generator for dynamics and hence conserved.

Using the imaginary part of the energy as generator for dynamics may seem odd. However, consider the following argument: For , the real part  is conserved in time, while we have no conservation law for . If the manifold  is accurate, we can expect  to remain small (assuming *H* = *H*^†^). On the other hand, for , the imaginary part  is conserved—and if  is accurate, it will be small—but it is now  that fluctuates, but should hopefully be *almost* conserved. In this sense, the two principles may be regarded as complementary, and reflect the ubiquitous compromise in bivariational theory, being “non-Hermitian” in nature.

In Section 5.2 it is proven that for a certain canonical extension of a univariational theory,  = 0 is equivalent to the TDVP, where energy is explicitly conserved, and  = 0 is equivalent to the MVP, where energy is *not* explicitly conserved. This lends credibility to the claim that for certain bivariational approximations,  is indeed a meaningful action. On the other hand, in Section 5.3 we also point out a slightly different extension of univariational theory is presented where it does *not* make sense to use .

This indicates that there could be mathematical criteria that distinguish bivariational manifolds into types where both  and  yield meaningful actions that in principle can converge to the exact result when  is increased in some as of yet undefined manner, and manifolds where either one or both of  or  become degenerate, either in the exact limit or identically. We briefly return to this discussion in Section 6.7, but we relegate a deeper analysis for future research.

## 4 Poisson Brackets and Conservation Laws

In this section, we study Poisson brackets and conservation laws for approximate bivariational evolution.

### 4.1 Poisson Bracket

Let  and  be smooth scalar-valued functions of , for the moment assumed to be a complex submanifold of . We define a Poisson bracket in local coordinates
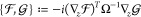
20which is again a smooth scalar valued function over . Due to the antisymmetry of ω
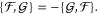
and it is also readily shown^5^ that the bracket satisfies the Jacobi identity
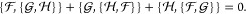


In the case where , the Poisson bracket takes the form
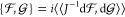
21which is coordinate-free. By using the representation (18) we obtain a coordinate-free formula for the Poisson bracket (20) on the complex manifold 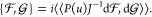
22

The Poisson bracket  generates time evolution. By the chain rule, it is seen that
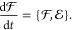
23

In particular, the coordinates themselves are smooth over , which gives
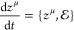


### 4.2 Ehrenfest’s Theorem

Recall that for an operator *A* on , we have defined its symmetrization *Â* on  as  = . We invite the reader to verify the following useful formulas: For all bounded operators *A*, *B*: , and for all *u*, *v* ∈ , *w* ∈ 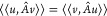
24a
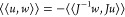
24band
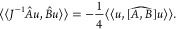
24c

We define the expectation value of *A* with respect to  ≡ *u* ∈  by
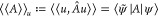


Let *u*(*t*) be a solution to eq 10. Then, using eq 24c and the Poisson bracket (21) with  = 2*Âu*, one obtains the following Poisson bracket
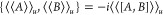


In particular, one obtains Ehrenfest’s Theorem on bivariational form
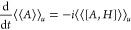
25

### 4.3 Conservation Laws on Complex Manifolds

We next present the generalization of the Bivariational Ehrenfest theorem to manifolds. Let  be a complex manifold. We say that *Âpreserves* if
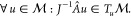


Let  be a solution to the bivariational principle on , and hence a solution to the -projected Euler–Lagrange equation, eq 12. Using eq 23, we see that
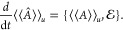


Applying eq 24b to the Poisson bracket formula in eq 22, and inserting  and , we get
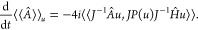


If *Â* preserves , then we can use the definition of *P*(*u*) in eq 13 together with eq 24c to obtain
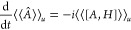


In general, {⟨⟨*A*⟩⟩_*u*_, ⟨⟨*B*⟩⟩_*u*_} = –*i*⟨⟨[*A*, *B*]⟩⟩_*u*_ if *Â* or *B̂* preserves . If *Â* does not preserve , it is possible to express the deviation from Ehrenfest’s theorem in terms of the distance between  and . One can thus obtain bounds for change in expectation value of observables. Indeed, we have in general
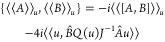
where *Q*(*u*) = 1 – *P*(*u*). Thus, for a preserved variable ⟨⟨*A*⟩⟩ evolving on a complex manifold, we obtain the bound of the error
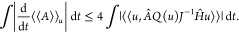
26

Such error bounds can be expressed in terms of the curvature of the manifold ,^5^ but we do not consider this further here.

### 4.4 Conservation Laws on Real Manifolds

The preceding discussion generalizes to the setting of real manifolds. Let  be a real manifold. Then, the Poisson bracket
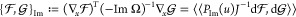
generates the time evolution of the real bivariational principle  on  by
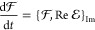
for any two scalar valued functions  and . Similarly, the Poisson bracket
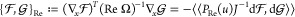
generates time evolution of the imaginary bivariational principle  on  by
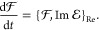


The corresponding equations for the time evolution of expectation values are given by
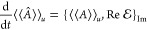
for the real bivariational principle and
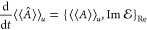
for the imaginary bivariational principle. This allows us to formulate a corresponding conservation law for expectation values. Indeed, for  = 0 we have for any two operators *A* and *B* that
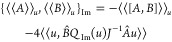
where *Q*_Im_(*u*) = 1 – *P*_Im_(*u*). Similarly, for  = 0 we have for any two operators *A* and *B* that
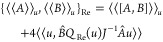
where *Q*_Re_(*u*) = 1 − *P*_Re_(*u*). We have corresponding conservation laws for operators which preserve , and error bounds corresponding to eq 26 apply for both  = 0 and  = 0.

## 5 Relation to Univariational Theory

### 5.1 TDVP vs MVP

The branching of the complex TD-BIVP into two distinct real bivariational principles is similar to the relationship between the TDVP and the MVP for *univariational* approximations of the Schrödinger equation.^4−6,30,39^ The TDVP recasts the TDSE as a principle of stationary action via the functional
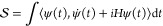
27where ⟨·, ·⟩ is the ordinary inner product. One must now assume that *H* = *H*^†^. Using this, it is straightforward to show that  = 0 if and only if *i*ψ̇ = *H*ψ. Suppose now  is a *real* submanifold. The stationary condition of  then becomes after using integration by parts
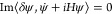
28for all .

If we define the symplectic form  =  on , then, eq 28 is equivalent to
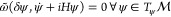


We may rephrase in terms of the symplectic projection , such that eq 28 is equivalent to
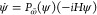
29

The McLachlan variational principle^3,5^ states that ψ̇ = Θ, where Θ is defined by
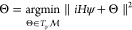


Differentiating ∥*iH*ψ + Θ∥^2^ with respect to Θ, equating to zero and inserting ψ̇ = Θ gives
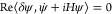
30for all . The form Re⟨ψ, ϕ⟩ is the inner product on the realification , so that eq 30 is equivalent to
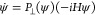
31with  being the orthogonal projection.

Suppose now  has the property that  ∈  ⇔  ∈  This happens if  is actually a complex submanifold of . Then eqs 28 and 30 are equivalent, and the symplectic and orthogonal projections coincide,  = .

We remark here, that the symplectic and orthogonal projections are very different mathematical operations. The orthogonal projection *P*_⊥_(ψ) appears since the MVP *minimizes the distance* between ψ̇ and *iH*ψ, i.e., *P*_⊥_(ψ)(−*iH*ψ) is the best possible approximation in the norm. In contrast, the symplectic projection  has *in general no similar interpretation in terms of distance minimization*.

The TDVP and the McLachlan principles are distinct for real manifolds, but equivalent for complex manifolds, where the symplectic projection and orthogonal projections coincide. We contrast this with the bivariational case, where the projections *P*_Re_(*u*) and *P*_Im_(*u*) are distinct for real manifolds, and *P*_Re_(*u*) = *P*_Im_(*u*) = *P*(*u*) for complex manifolds. Notably, in all the latter cases we deal with the symplectic projection.

### 5.2 Bivariational Generalization of TDVP and MVP

In the univariational time-dependent principles, an evolution ψ(*t*) on a manifold  is sought that in some sense is as close as possible to the exact evolution generated by a self-adjoint Hamiltonian *H*. Using MVP, orthogonal projection onto  is used, while using TDVP symplectic projection is used. Suppose now, that *H* is no longer self-adjoint. Then, the natural generalization would be to let  =  and  be independent variables, i.e., appeal to the TD-BIVP using the bivariational manifold . Indeed, one of the first applications of bivariational theory on this form was proposed by Froelich and Löwdin^40^ for (time-independent) Hartree–Fock theory. When *H* is actually self-adjoint, the TD-BIVP using the manifold  should reduce to univariational theory. Indeed, one can show that in this case, the TDVP *and* the MVP can be derived from the real and imaginary bivariational principles, respectively.

To be precise, we will show the following: If we pick an initial condition on the form  where  denotes the injection from  to ,^1^ and assuming *H* is self-adjoint, then the solution to the variational principle  = 0 equals *u*(*t*) = , where ψ(*t*) is the solution to the TDVP, with initial condition ψ_0_. Likewise, the solution to  with initial condition *u*_0_ corresponds to ψ(*t*) being a solution to the MVP.

First, observe that for arbitrary variations of the bivariational action, we have
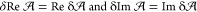


Writing out the expression for  from eq 8, we have
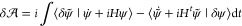


Thus,  reads
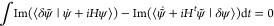
32for all  and . Equation 32 is equivalent to
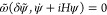
33afor all  and
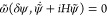
33bfor all δψ ∈ *T*_ψ_*M*. By using the definition of  and , we see that eqs 33a and 33b are equivalent to
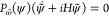
34aand
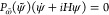
34brespectively.

By selecting equal initial conditions for ψ̃ and ψ, eq 34a and 34b give equal time evolution for ψ̃ and ψ. We recognize this to be the one given by the TDVP.

By an analogous argument, setting  gives
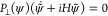
and
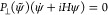
which recovers MVP when we select equal initial conditions for ψ̃ and ψ.

It is interesting to observe, that our derivation implies that the MVP can be associated with a sympletic projection, namely *P*_Re_(*u*) defined in Section 3.2, and we can also use the corresponding Poisson bracket {·, ·}_Re_ from Section 4.4 to study the MVP dynamics.

### 5.3 Variable Reduction

We now show how the manifold  can be reduced to the “diagonal”
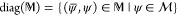
a manifold of *half* the dimension of , since the “bra” and “ket” are no longer independent.

A straightforward calculation shows that on this manifold , the TDVP action from eq 27, and in particular
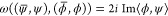
replicating the symplectic structure on . Since the TDVP action is real-valued, the TDVP is equivalent to , while on the other hand , implying that the imaginary TD-BIVP is degenerate for this particular manifold, i.e., it will not give meaningful equations of motion. The MVP is thus not reproduced.

The present reduction of  is relevant, since various practical TDCC methods are actually defined in terms of such reductions of complex bivariational manifolds, e.g., the TD-ooCC family of methods to be described in Section 6.3. For these methods, therefore, we predict that  is risky business, and that  may be the preferred option in most cases.

## 6 Unifying View of Current Time-dependent Bivariational Approaches

The abstract formalism in this article is applicable to many methods for real-time propagation encountered in the literature. In this section, we give a brief overview of what we consider to be some important examples in the language of the present work, all being varieties of coupled-cluster (CC) theory: Time-dependent traditional CC theory (TDCC), orbital-adaptive time-dependent CC (OATDCC) theory (known as time-dependent modal vibrational CC (TDMVCC) theory when applied to vibrational dynamics), orbital-optimized time-dependent CC (TD-ooCC) theory, including time-dependent optimized CC (TD-OCC) and the split time-dependent modal vibrational CC method (sTDMVCC), time-dependent Brueckner CC (TD-BCC) theory, and finally time-dependent equation-of-motion CC (TD-EOM-CC) theory.

Familiarity with CC theory is assumed, and the presentation emphasizes the bivariational manifold structure of the methods. We refer to the original publications for full details. For a recent review of real-time propagation with CC theory, see ref (22). For a recent overview of TD-ooCC methods, see ref (32).

### 6.1 Traditional CC Ansatz

The traditional CC method is the most popular wave function-based method for electronic-structure theory, with the CCSD(T) model often termed “the gold standard of quantum chemistry” due to its balance of cost and accuracy.^41^ Similarly, the CC method offers an attractive approach for approximating the solution to the vibrational Schrödinger equation.^42^ Although the physical nature of the degrees of freedom and of the Hamiltonian is very different in the electronic and vibrational cases, both cases benefit from fast convergence of the CC hierarchy, polynomial-scaling cost, and size extensivity.

The traditional CC method is usually formulated in finite-dimensional subspace of , defined in terms of a finite orthonormal set of single-particle functions (a “basis set”). However, we here take a broader picture, and merely assume a *biorthogonal* set of single-particle functions partitioned into occupied and unoccupied subsets,  = , and  = . This induces a biorthogonal many-particle basis set  and  with  =  being formal excitation references for bras and kets, respectively. The full configuration-interaction wave function and its dual are written ψ = Cϕ and , with *C* =  and *C̃* =  being cluster operators (the summations run over μ ≥ 0). Here, *X*_μ_ϕ = ϕ_μ_ is an elementary excitation, and similarly  is an elementary dual excitation, or de-excitation. Note that *X*_0_ϕ = ϕ and , i.e. *X*_0_ and  are simply identity operators or null excitations. The notation *A*^*t*^ is defined via the dual pairing ⟨*A*^*t*^ϕ|ψ⟩ ≡ ⟨ϕ|*A*ψ⟩. Equivalently, using bra notation, .

The space  is an abelian algebra that depends on the subspaces  =  only, and not on the individual basis functions. Similarly,  = . The spaces  and  are dual to each other, with the dual pairing being given by  = .

In traditional CC theory, the phase-space point  is parametrized in terms of a pair of cluster operators  ∈  as
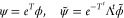
35where *T* = ∑_μ_τ_μ_*X*_μ_ and . Again, μ = 0 is included in the summations. τ_0_ plays the role of a phase/norm factor, while λ_0_ determines the intermediate normalization in the sense . Equation 35 defines a map  being a global coordinate chart for a smooth complex submanifold . In fact, this submanifold covers *almost* all possible points in . The additional conditions are  and .

The energy functional in these coordinates is
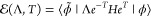
the conventional CC Lagrangian (which is a Lagrangian in the sense of constrained optimization, and must not to be confused with the Lagrangian density encountered in the bivariational principle), and the action functional reads
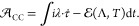
36

(The integrand is the Lagrangian density in our language.) In particular, the functional form is preserved compared to eq 1. This means, that the coordinate transformation  is a canonical transformation in the sense of classical mechanics, and it follows that Hamilton’s equations of motion (both the complex and real forms) are preserved as well.

The full CC case is not practical, and conventional truncation schemes  of the cluster operators imply an approximate submanifold . For electronic-structure theory,  is typically the CCSD···*K* scheme, where all excitations of up to *K* electrons are included. In the vibrational case, the analogous approach is usually denoted VCC[*K*] and includes up to *K*-mode excitations. Since the coordinates are canonical, the induced symplectic form on  is trivially nondegenerate, and the submanifold is always symplectic.

Since the coordinates (Λ, *T*) are canonical, the Poisson bracket takes on the simple form
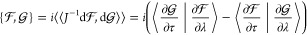


For expectation values,  = , we have  = , where  = , and  = . Evaluation of the Poisson bracket yields
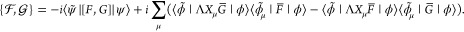


In particular, if we set , we obtain that  is exactly conserved under dynamics only if the last two terms vanish.

### 6.2 OACC Ansatz

The CC ansatz described above is defined in terms of a *static* single-particle basis. In particular, the references  are static, which is a serious limitation in terms of describing, say, large oscillations in the wave function, or motion far away from the ground state. The traditional CC ansatz works well when the amplitudes are sufficiently small, i.e., when the reference describes a large part of the wave function. Conversely, if the wave function moves too far from the reference, the amplitudes grow and the ansatz tends to break down. The paradigmatic example of such a situation is ionization or dissociation, i.e. the removal of one or more particles from the system (typically by a laser pulse). However, much less violent phenomena can also initiate the breakdown of the CC ansatz, as exemplified in vibrational CC theory by the internal vibrational energy redistribution (IVR) in water.^16^

This problem can be alleviated by introducing an *adaptive* single-particle basis: Both the occupied and unoccupied single-particle bra and ket basis functions are time dependent. This in turn defines a pair of adaptive references *and* excited determinants that move with the wave function, so to speak.

We now consider the general structure of the manifold of CC states  when the single-particle basis is allowed to vary. We first note that the individual basis functions do not matter, only the subspaces spanned by these.

We have a splitting  = , where  is the part of single-particle space not captured by the finite basis and not part of the state. Similar statements hold for the “bra” bases and spaces. In particular, . Additionally, biorthogonality means that , , etc. However, it is not true in general that , etc., since the bases are not orthonormal.

The cluster operator algebras are now functions of the unoccupied and occupied spaces:  = , and  = . Thus, the bivariational CC manifold depends on the choice subspace splittings: , *U* =  denotes the tuple of single-particle spaces. The set of all such space selections is denoted , and is a differentiable manifold. Restricting cluster operators to a certain truncation , see Section 6.1, again leads to a submanifold .

In traditional CC, the manifolds are *fixed*, but the idea of OATDCC is to let the spaces—or in practice their basis functions—be time dependent. Thus, one seeks not only Λ(*t*) and *T*(*t*), but also *U*(*t*).

The complex orbital-adaptive CC manifold is thus defined by
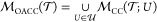


In practice, this is an immersed manifold—it can have self-intersections.

It has been found that singles excitations must be removed from  in order to produce a well-defined manifold.^15^ The manifold  is a complex manifold due to the independence of the bra and ket single-particle functions.

The orbital-adaptive time-dependent coupled-cluster (OATDCC) ansatz was first introduced by Kvaal^15^ for describing electron dynamics. An analogous ansatz for the vibrational problem was proposed by Madsen et al.^16^ under the name time-dependent modal vibrational coupled-cluster (TDMVCC). The formulation by Kvaal is done in the above abstract infinite-dimensional setting, but, for simplicity, we consider here a finite basis and use the exponential parametrization of the manifold  of single-particle subspaces. To that end, given an arbitrary *U* =  ∈ , any *other* can be written as
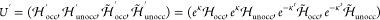
here, κ is a generic (neither Hermitian nor anti-Hermitian) one-particle operator. Introducing the creation and annihilation operators associated with the biorthogonal single-particle basis functions for the full single-particle space , the corresponding second-quantized operator is
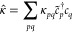


In fact,  and  are change of single-particle basis operators. Correspondingly, for any  ∈ , we can apply the basis change operators and obtain *every* element
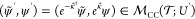


However, the parametrization contains redundancies that must be eliminated or fixed by a suitable gauge condition. The source of the redundancy is the invariance of the CC wave functions under mixings of occupied single-particle functions and unoccupied single-particle functions separately. Thus, one allowed gauge condition (at κ = 0) is to set all elements of κ to zero that mix occupied, unoccupied, or secondary/virtual single-particle functions separately, and keep the rest, i.e., those that mix the different types of single-particle functions. The mathematical structure is that of a principal bundle.^5^

It is instructive to consider the Lagrangian, i.e. the integrand of the action, eq 1
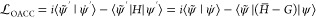
here,  and *G* = *ie*^–κ̂^(d*e*^κ̂^/d*t*) = −*i*(d*e*^–κ̂^/d*t*)*e*^κ̂^.  is in fact identical to the integrand of the action in traditional CC theory, cf. eq 36, provided we substitute *H* ← *H̅* – *G*, so the AOCC amplitude equations have the same form as the CC amplitude equations.^15,20^ Stationarity of  leads to a set of linear equations for *G*, which in turn determines κ̇. At the point κ = 0, the relation is particularly simple, namely *G* = *i*κ̇ (the κ ≠ 0 case has been treated in detail in ref (43)). We remark that refs (15 and 20) did not use the exponential parametrization for the single-particle basis, but the linear equations are the same.

A special case of the OACC ansatz is obtained if the spaces  and  are empty, i.e., the spans of the single-particle bases  and  are fixed. Equivalently, there is no *secondary space*, and the CC parametrization is allowed to correlate all available single-particle functions. In this case, the OACC formalism becomes formally equivalent to nonorthogonal orbital-optimized CC theory (NOCC).^44^ For the full derivation of OATDCC see ref (15).

### 6.3 Orthogonal Optimized CC Ansatz

The OATDCC approach introduces a complex differentiable ansatz manifold at the cost of having two sets of time-dependent single-particle functions. A class of methods that instead use *orthonormal* single-particle functions is the time-dependent orbital-optimized CC (TD-ooCC) family of methods discussed by Sato and co-workers.^31,32^ These methods can be viewed as constructed from the OACC ansatz by restricting this manifold to a “diagonal” analogous to the construction in Section 5.3: The biorthogonal subspaces *U* =  are restricted to being on the form  = , where additionally . Thus, the biorthogonal decomposition is reduced to an orthogonal decomposition  = . Letting *V* =  denote a selection of subspaces, we obtain , cf. the previous subsection. We obtain a bivariational submanifold
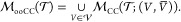


The action principle  is employed to obtain equations of motion. Could one appeal to the alternative principle ? In the limit of a complete CI expansion in the active space (i.e., CASCCF), full CC and CI are equivalent, the physical solution satisfying *u*(*t*) = (ψ(*t*)^†^, ψ(*t*)). For orbital variations from the active space to the secondary space alone, a quick calculation shows that Re ω(δ*u*, δ′*u*) ≡ 0 on the physical shell, where δ′*u* is a second arbitrary variation. Thus, the block of the equations of motion derived from  that correspond to such active-secondary orbital rotations become singular. For variations in the active space, the situation is more unclear, and further study is needed. In any case, at least when a secondary space is present, it seems that the principle  for TD-ooCC manifolds becomes ill-behaved.

In the OATDCC formalism, the singles amplitudes of τ_1_ produce nonorthogonal orbital rotations and are therefore redundant. For TD-ooCC, however, the orbital rotations allowed are constrained to be orthonormal. The singles amplitudes of λ and τ are thus not exactly redundant, but nevertheless may introduce numerical instabilities. In ref (32), different methods are obtained from the various possible choices of using the singles amplitudes as variational parameters or constraints. In the time-dependent optimized CC (TD-OCC) method, all singles amplitudes of λ and τ are constrained to be zero. Thus, compared to the OATDCC method, the orbital rotations are constrained to be orthonormal, rather than biorthogonal.

The CC amplitudes λ and τ (and their complex conjugates) appear in a complex differentiable manner in the OCC ansatz, so  leads to amplitude equations with the same structure as for . The TD-OCC and OATDCC *amplitude* equations are thus essentially identical in structure as the time-dependent traditional CC amplitude equations. The linear equations that determine the OCC basis set evolution are, however, symmetrized in TD-OCC compared to the OATDCC equations. As an example, the density matrices that appear in the OATDCC working equations are Hermitianized in the TD-OCC working equations (cf. refs (15, 31 and 32)).

### 6.4 Brueckner CC Ansatz

In CC theory, a common problem is that large amplitudes lead to large non-Hermiticity of the state . A mitigation would be to choose the Brueckner basis: For a wave function , the Brueckner reference determinant is defined by the criterion that ⟨ϕ|ψ⟩ is maximal, i.e.,  for all single excitations. Given  this differential condition thus specifies the splitting . However, the single de-excitation amplitudes λ_*a*_^*i*^ are *not* fixed by this choice. Thus, the time-dependent Brueckner CC (TD-BCC) method is defined as a TD-ooCC method that uses the truncation scheme  containing up to *K*-fold excited λ-amplitudes *including* singles amplitudes, and up to *K*-fold excited τ-amplitudes *except* singles amplitudes. We obtain the following *real* bivariational manifold
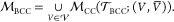
37

Relative to TD-OCC, λ_*a*_^*i*^ are *added* as bivariational variables. As member of the ooCC family,  is used to derive equations of motion.

Optimizing over this manifold in the time-independent case is equivalent to a method introduced by Köhn and Olsen,^45^ which argue that it reproduces FCI in the limit of no cluster operator truncations.

### 6.5 TD-OCCT1 and sTDMVCC ansätze

Whereas TD-BCC included *λ*_*a*_^*i*^ as bivariational parameters, it is also possible to instead choose τ_*i*_^*a*^, leaving λ_*a*_^*i*^ ≡ 0. The single-particle functions are still required to be orthonormal. This leads to a bivariational manifold dubbed the OCCT1-ansatz by Lang and Sato^32^
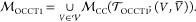
Here,  is defined by having the τ singles included, while the λ singles are not included.

Interestingly, it turns out that TD-OCCT1 is gauge equivalent^32^ to the split-TDMVCC (sTDMVCC) ansatz recently introduced by Højlund and Christiansen^33^ for the vibrational Schrödinger equation. Notably, this ansatz is defined as a reduction of OATDCC, and *not* TD-ooCC. The sTDMVCC approach was motivated by the following observation: The active “bra” and “ket” spaces tend to drift far apart in TDMVCC/OATDCC calculations, leading to strong non-Hermiticity of the state , and hence inaccurate results and numerical instabilities. The proposed solution is to enforce that the -spaces are always explicitly orthogonal, i.e., , *while allowing the active single-particle functions to be biorthogonal and otherwise general*. Thus, since , it follows that  = . Working in the exponential parametrization of the single-particle functions, the authors posited a factorized form
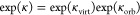
38here, κ_orb_ is general and nonanti-Hermitian, generating all possible rotations between occupied and unoccupied orbitals, while κ_virt_ is explicitly anti-Hermitian, and only changes the total space  in a unitary manner. Thus,  is preserved.

Lang and Sato proved that TD-OCCT1 and sTDMVCC are in fact equivalent. We can make the interesting and somewhat surprising observation that sTDMVCC is gauge-equivalent to a method where *all* single-particle/single-modal functions are orthonormal, even though τ_1_ effectively changes these orthonormal functions to biorthonormal ones. It must be mentioned, that the equivalence holds in exact arithmetic. The methods may behave differently in finite arithmetic, and depending on time integration methods.

Finally, we note the formal equivalence of OATDCC, TD-OCCT1, and TD-NOCC, observed by Lang and Sato,^32^ when there is no secondary space.

### 6.6 Time-dependent EOM-CC

Equation-of-motion CC theory is perhaps the simplest example of a bivariational method where non-Hermiticity is explicit. In EOM-CC, a ground-state calculation is first performed with traditional CC theory, producing a cluster operator  (and also a ) such that ψ_0_ = *e*^*T*_0_^ϕ is an approximate ground state. Using the theory of linear response, excited-state energies are approximated by the eigenvalues of a “dressed”, or effective Hamiltonian, *A* = *Pe*^–*T*_0_^*He*^*T*_0_^*P*, where *P* is the orthogonal projector onto the configuration-interaction space, usually at the same level of truncation  as the underlying CC calculation. Thus, a bivariate Rayleigh quotient is set up,  = , where . The left and right eigenvectors of *A* are treated as approximations to excited states. The action of TD-EOM-CC theory is simply
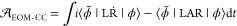
producing linear canonical equations of motion and a simple Poisson bracket, formally identical to eq 21. Since this action is complex differentiable, the actions , , and  lead to identical equations of motion.

### 6.7 Two Types of Bivariational Manifolds

Given a real bivariational manifold , can we determine whether both  and  are meaningful actions? In light of the construction in Section 5.2, one could then expect the two principles to approximately correspond to the TDVP and MVP, including their geometric interpretations in terms of symplectic and orthogonal projections, respectively. We will not present definite answers, but instead compare the approach of OATDCC/TDMVCC and the TD-ooCC family of methods in a qualitative manner.

In OATDCC/TDMVCC, introducing biorthogonal subspaces can be viewed as a generalization of the idea of Froelich and Löwdin^40^ who introduced biorthogonal single-particle functions for non-Hermitian Hartree–Fock before invoking the bivariational principle. The generalization consists of letting all originally univariational variables (the single-particle functions) be promoted to independent variables for the “bra” and the “ket” before invoking the TD-BIVP. Thus, the construction of  is analogous to the promotion of a univariational approach with manifold  to the bivariational manifold  = , as described in Section 5.2. In particular  is a *complex* manifold, such that  and  are equivalent actions.

Suppose we now introduce a submanifold by placing restrictions on *U* = , where *V* =  and *Ṽ* = . For example, we could introduce real submanifolds, such as Gaussian functions with variable centers and widths for all the four spaces separately (Whether this particular choice will “work” is not the issue here.) We then obtain a *real* submanifold , and the real and imaginary parts of the bivariational action are *no longer equivalent*. However, in this case, the restriction is done in a manner such that the “bra” and “ket” parts of the variables are still independent. Thus, in this situation we expect  to correspond to a bivariational version of the TDVP, while  should correspond to bivariational version of the MVP. In particular, both should be meaningful.

Let us contrast this with the TD-ooCC family of methods. Here, the restriction of the bivariational variables is done in a different manner. We have that  is restricted to a “diagonal” by enforcing , and additionally that . This situation is similar to the transition from  =  to  in Section 5.3, where only  was a meaningful action. Thus, for TD-ooCC we expect  still to correspond to a bivariational version of the TDVP, while  may in some cases be very close to a degenerate functional, a topic we relegate to future studies.

Note that we are *not* concluding that the TD-ooCC family is somehow an inferior method due to  probably being problematic in some cases. On the contrary, we offer a qualitative insight into the fact that the very construction of TD-ooCC seems intrinsically linked with TDVP as opposed to the MVP.

## 7 Conclusion

In this article, we studied the time-dependent bivariational principle, and employed a differential geometric point of view. We introduced an action principle , where the field variables are the wave function and its complex conjugate . Approximate propagation techniques of bivariational type are then obtained by restricting these to lie in a smooth submanifold, or ansatz space.

We demonstrated that taking the real and imaginary parts  and  resulted in two independent variational principles that both reproduce exact dynamics when no approximations in the wave functions are introduced. A distinction was further made of approximate methods depending on the ansatz space being parametrized with complex or real coordinates. When complex coordinates are used, the two variational principles are equivalent. When real coordinates are used, the real and imaginary principles are not always equivalent. Comparison with the time-dependent (uni)variational principle and the McLachlan variational principle were made, and it was demonstrated how both these principles can be formulated using a real bivariational manifold.

In analogy with classical mechanics, Poisson brackets were introduced that allow analogy with the transition from classical to quantum mechanics. In particular, time evolution of observables become Poisson brackets.

The physical meaning and applicability of  for real bivariational manifolds is presently unclear, since the generator for time evolution is then not the real part of the energy, but instead the imaginary part. It is not from the outset “unphysical”, since bivariational methods invariably introduce formally complex-valued energies and expectation values. However, it was demonstrated that the TDVP is equivalent to  while the MVP is equivalent to  for a particular but natural bivariational generalization of a univariational manifold. A second construction, using a reduced set of bivariational variables, revealed how  becomes degenerate in this case.

In the final section of the article, we formulated various methods for real-time propagation using the TD-BIVP. We formulated time-dependent traditional coupled-cluster theory, orbital-adaptive time-dependent coupled-cluster theory and various orbital optimized coupled-cluster methods, where the single-particle functions are allowed to move during dynamics, as well as time-dependent equation-of-motion coupled cluster theory. The orbital-adaptive and orbital-optimized methods were compared with respect to whether  is a useful principle or not. This discussion was linked to the above-mentioned comparison of the TDVP/MVP and the TD-BIVP.

## Appendix

### Time-independent bivariational principle

In this section, let . Consider initially the stationary bivariational principle: Let  be a (not necessarily self-adjoint) operator, let , and consider the bivariate Rayleigh quotient

39

The functional  is a bivariational expectation value functional for the operator *A*. It is a Fréchet smooth function, which means that it can be Taylor expanded in its argument about any point. Infinitesimal variations  are expressed as directional derivatives, and  for all variations δu if and only if , if and only if

40

where . Here,  can be written using bra notation as . Thus, *both* left and right eigenvectors for the eigenvalue *a* must exist for a critical point to exist. This clearly restricts the class of non-selfadjoint operators that can be treated, but the main point is that *A* being Hermitian was *not* used in the proof of the bivariational principle, in contrast to the corresponding proof for the standard Rayleigh–Ritz variational principle. Instead, we are given the choice to approximate ψ̃ and ψ with *independent* approximations.

We observe that  is invariant under individual phase and normalization transformations on , that is  = , for any . Indeed, we can express , where , which can be thought of as a pure state in the bivariational setting. Thus, we want *all physical predictions* to be stated in terms of ρ.

The scale invariance of ρ implies that the critical point condition (40) does not yield locally unique solutions: For every critical point , another critical point is given by  for any nonzero . Moreover, if *a* is not a simple eigenvalue (meaning that *a* is a degenerate eigenvalue), there will be further degrees of freedom in the set of critical points.

### Time-dependent bivariational principle in terms of the density operator

The development in the present section is similar to the treatment of the time-dependent variational principle in ref (4). Consider the integral

41

This functional is invariant (up to a total time derivative) under *time-local* phase and normalization transformation of ψ̃(t) and ψ(*t*) individually, and hence the critical point histories (solutions of the Euler–Lagrange equations) are unique only up to such changes. On the other hand, the time-dependent state ρ(*t*) is unique. Denote the integrand in  by . The Euler–Lagrange equations are equivalent to

42

and

43

For every solution, *u*(*t*), new solutions are generated by time-local phase and normalization transformations . These leave ρ(*t*) invariant. One particular set of solutions is the “canonical” solutions to the time-dependent Schrödinger equation and its dual

44

One can see that all other solutions can be mapped to a solution on this form via a particular local phase and normalization transformation. It follows that all solutions with the same initial condition up to phase and normalization are equivalent to the canonical solution. Thus,  is the action functional  from Section 2 expressed in terms of the density operator ρ(*t*). Indeed, the canonical solutions to  are precisely the solutions to .

Consider now complex or real submanifold . For  to be equivalent to , we have two sufficient conditions: (1)  is overlap normalized, i.e., . Indeed, then  and  become identical functionals. (2)  contains “rays”, i.e., if , then  for all . Indeed, then  is still invariant under time-local phase and normalization changes. However, we observe, that for such a manifold, we can select a submanifold on the form (1) that will generate the same solutions using the functional .

## Footnotes

1 The injection is not the complex conjugate, which does not exist for general complex Hilbert spaces, but rather a “renaming” of ψ so that it lies in . The vector itself is not changed.^34^
